# Knowledge of malaria prevention among pregnant women and non-pregnant mothers of children aged under 5 years in Ibadan, South West Nigeria

**DOI:** 10.1186/s12936-019-2706-1

**Published:** 2019-03-22

**Authors:** Kelechi Elizabeth Oladimeji, Joyce Mahlako Tsoka-Gwegweni, Elizabeth Ojewole, Samuel Tassi Yunga

**Affiliations:** 1 Department of Public Health, College of Health Sciences, University of KwaZulu-Natal, Durban, South Africa; 2 Faculty of Health Sciences, University of Free State, Bloemfontein, Free State South Africa; 3 Discipline of Pharmaceutical Sciences, School of Health Sciences, College of Health Sciences, University of KwaZulu-Natal, Durban, South Africa; 4 Department of Tropical Medicine, Medical Microbiology and Pharmacology, John A. Burns School of Medicine, University of Hawaii at Manoa, Honolulu, USA; 5 The Biotechnology Center, University of Yaoundé 1, Yaoundé, Cameroon

**Keywords:** Malaria prevention and control, Pregnant women, Non-pregnant mothers of children aged under 5 years

## Abstract

**Background:**

Adequate knowledge of malaria prevention and control can help in reducing the growing burden of malaria among vulnerable groups, particularly pregnant women and children aged under 5 years living in malaria endemic settings. Similar studies have been conducted but with less focus on these vulnerable groups. This study assessed knowledge of malaria prevention and control among the pregnant women and non-pregnant mothers of children aged under 5 years in Ibadan, Oyo State, South West Nigeria.

**Methods:**

In this cross sectional study, data on socio-demographic, clinical and knowledge on malaria prevention was collected using interviewer administered questionnaires from consenting study participants attending Adeoyo maternity hospital between May and November 2016. Data was described using percentages and compared across the two maternal groups in the study population. Knowledge scoring from collected data was computed using the variables on causes, symptoms and prevention of malaria and thereafter dichotomised. Multivariate analyses were used to assess the interactive effect of socio demographic and clinical characteristics with malaria knowledge. Level of statistical significance was set at p < 0.05.

**Results:**

Of the 1373 women in the study, 59.6% (818) were pregnant women while 40.4% (555) were mothers of children aged under 5 years. The respondents mean age was 29 years ± 5.2. A considerable proportion of both the pregnant women (n = 494, 60.4%) and the non-pregnant mothers of children aged under 5 years (n = 254, 45.8%) did not have correct knowledge on malaria prevention measures based on our assessment threshold (p < 0.001). Having a tertiary level education was associated with better knowledge on malaria (4.20 ± 1.18, F = 16.80, p < 0.001). Multivariate analyses showed that marital status, educational attainment, gravidity, and HIV status were significantly associated with knowledge of malaria prevention and control.

**Conclusion:**

The findings indicate that socio-demographic factors such as marital and educational status greatly influence knowledge on malaria prevention and control measures. Key health stakeholders and authorities need to implement strategies and direct resources to improve the knowledge of mothers on malaria prevention and control. This would stem the tides of malaria related deaths among pregnant women and children aged under 5 years.

## Background

Malaria is a major public health problem in ninety-one countries world-wide with sub-Sahara Africa bearing 80% of the disease burden [1]. Malaria remains endemic in Nigeria where the parasitic disease disproportionately affects children aged under 5 years and pregnant women compared to the rest of the population groups [2–6]. In pregnancy, malaria increases the risk of maternal anaemia, spontaneous abortions, stillbirths, premature deliveries, intra-uterine growth retardation and low birth weight babies, and these are all important causes of infant mortality [7]. Also, more than 70% of all malaria deaths occur in children aged under 5 years [4, 8]. The scope of malaria control is changing worldwide with more emphasis on community and individual participation. Health education can improve participation in malaria control, when such education is designed to address gaps in the knowledge, attitudes and practice of individuals in the communities [4, 9]. Nigeria has implemented three national malaria strategic plans (NMSP) till date, and is presently implementing a fourth NMSP (2014–2020). This fourth NMSP aims to achieve pre-elimination status and reduce malaria-related deaths to zero by 2020 [10].

Evidence from malaria knowledge, attitudes, and practices (KAP) studies reported that misconceptions on malaria transmission and risk factors still exist with adverse impact on malaria control programmes [11, 12]. Findings from a study conducted by Singh et al. in rural areas of Northern Nigeria revealed that although knowledge about malaria prevention measures was high (90%), it was poorly reflected in their practices (16%) [13]. Another study by Adebayo et al. [14] assessed the knowledge of malaria prevention among mothers of children aged under 5 years and pregnant women in a rural community in Southwest Nigeria. This latter study also found poor knowledge and utilization of malaria prevention measures among majority of the caregivers in the rural study area [14]. Considering the vulnerability of both children aged under 5 years and pregnant women to malaria [10, 15], this study aimed to determine the knowledge of malaria prevention and management among pregnant women and non-pregnant mothers of children aged under 5 years seeking health care at one of the main secondary maternity hospitals in Ibadan, Nigeria. Only few studies have assessed knowledge on malaria prevention among mothers in hospital-based setting. This study sought to fill this gap and provide new insights on the depth of knowledge gaps. The findings will help to improve implementation of integrated malaria control strategies. It will also be essential in establishing epidemiological and behavioural baseline indicators to evaluate and improve progress by malaria control programmes.

## Methods

### Ethics statement

Prior to data collection, ethical approval was obtained from the Oyo state ministry of health ethics committee (IRB AD13/479/1035) in Nigeria and from the biomedical research ethics committee (BREC- BE199/16) of the University of KwaZulu-Natal, South Africa. Study participants voluntarily signed written informed consent forms without any incentives. They consented because they believed their responses would contribute to increased effective control of malaria. The participants were also assured of confidentiality. The data collection tool was translated to both Yoruba, which is the dominant local language, and English language.

### Study design and setting

**Fig. 1:**
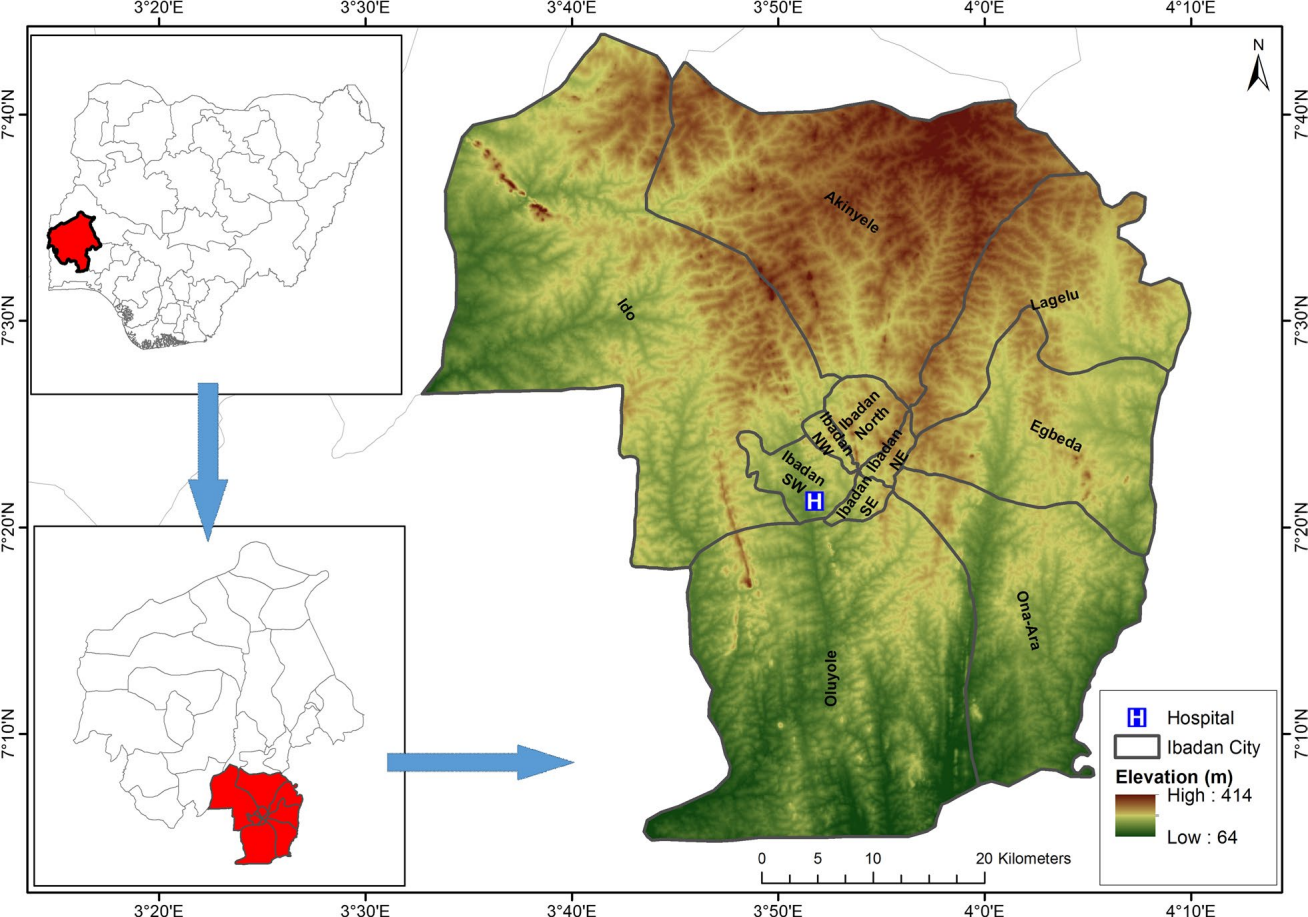
Elevation map showing Adeoyo Maternity Hospital, Ibadan

Using a cross sectional study design, this survey was conducted between May and November 2016. The study recruitment site was the Adeoyo Maternity Hospital located in Ibadan North East-Oyo state, Nigeria. The elevation of the study area lies between 64 and 414 mm (Fig. 1). The study setting and site have been described in another publication [16]. The hospital is situated in the semi-urban community of Yemetu-Adeoyo in Ibadan. This facility is one of the oldest of its kind in Nigeria (opened in 1927) that provides both primary and secondary level maternal and child health care [17].

### Study population and eligibility criteria

A multi stage sampling technique was employed with the aim of ensuring that the study population was representative of pregnant women and non-pregnant mothers of children aged under 5 years in the study area. The first stage involved identification of the geographical area and the second stage involved selection of the specific health facility from a list of facilities within the identified geographical area. In the third stage, participants were randomly selected from the selected health facility. The study population included consenting pregnant women and mothers of children under 5 years old attending the study site for health care. Mothers who were residents in Ibadan and regular attendees of the study site for health care were eligible to participate in the study. Criteria for inclusion into the study was that the women had to be either pregnant or have at least one child who is less than 5 years old.

### Data collection

A semi-structured interviewer administered questionnaire was used to collect data from the consenting study participants. The variables and measurements collected included socio demographic data such as age, socio-economic status; clinical characteristics such as human immunodeficiency virus (HIV) status, gravidity status, blood group; and questions assessing the participants’ awareness and extent of knowledge on malaria symptoms, prevention and management.

### Data analysis

Overall knowledge score was computed by aggregating the knowledge related variables (1) awareness of malaria (2) knowledge of cause of malaria (3) knowledge of breeding sites for mosquito (4) knowledge of three or more symptoms of malaria (5) knowledge of when malaria mosquito feeds (correct knowledge when at night), and (6) knowledge of malaria prevention knowledge (which include chemoprophylaxis, insecticide treated nets (ITN) and environmental sanitation). The knowledge variables were recoded to binary level such that respondents with correct option in the knowledge variables were coded 1 while not having correct knowledge was coded 0. Knowledge score was computed as the sum of the six knowledge variables, with 0 as the least possible score and 6 as highest possible score. Increasing score indicated better malaria knowledge. Subsequently, the median of the composite score was used as the cut-off to classify knowledge level as either poor or good. Individuals who scored less than the median of knowledge score were categorized as having poor knowledge while scoring within the exact median cut off and above were classified as having good malaria knowledge.

Categorical variables were presented as numbers and percentages; numerical variables were presented as means and standard deviation to describe the study population by their socio demographic and clinical characteristics. To assess the level of relationship and interaction between malaria knowledge score and the respondents’ socio demographic and clinical characteristics, analytical statistics involving Chi square and analysis of variance was carried out. Multivariate linear analysis was further performed to determine predictors of malaria knowledge. Level of statistical significance was set at p < 0.05. Analyses were performed using Statistical Package for the Social Sciences software (SPSS) version 25, Chicago, IL.

## Results

Table 1 presents results on the socio-demographic and clinical characteristics of the study respondents. Of the 1373 women in the study, 59.6% (818) were pregnant women whereas 40.4% (555) were non-pregnant mothers of children aged under 5 years. Mean age of respondents in the study was 29 years ± 5.2 years old. Mean age of the pregnant women in the study was 28.9 ± 5.21 while mean age of non-pregnant mothers of children aged under 5 years was 30.0 ± 5.14. The most predominant age group was 25–34 years of age (pregnant women: 71.3% vs non-pregnant mothers of children aged under 5 years: 66.8%). The most predominant socio economic class among both maternal groups were the lower upper class (60.4% for the pregnant women and 61.4% among non-pregnant mothers of children aged under 5 years). Married respondents were the majority in the study across both maternal groups (pregnant women: 89.4% vs non-pregnant mothers of children aged under 5 years: 95.5%). A larger proportion of the mothers had attained secondary education more than the less educated mothers, and this distribution was similar in both maternal groups (Table 1).

**Table 1:** Socio-demographic and clinical distribution by maternal group

	Maternal group		Total N (1373)
	Pregnant women N (%)	Non-pregnant mothers of children aged under 5 years N (%)	
Age group			
< 24	128 (15.6)	79 (14.2)	207
25–34	583 (71.3)	371 (66.8)	954
35+	107 (13.1)	105 (18.9)	212
Socio-economic status			
Lower class	140 (17.2)	62 (11.2)	202
Lower middle class	119 (14.6)	100 (18.0)	219
Lower upper class	492 (60.4)	341 (61.4)	833
Upper class	63 (7.7)	52 (9.4)	115
Marital status			
Never married	30 (3.7)	12 (2.2)	42
Married	731 (89.4)	530 (95.5)	1261
Separated/widowed	57 (7.0)	13 (2.3)	70
Education			
No formal education	76 (9.3)	21 (3.8)	97
Primary	40 (4.9)	41 (7.4)	81
Secondary	384 (46.9)	325 (58.6)	709
Tertiary	318 (38.9)	168 (30.3)	486
Religion			
Christianity	338 (41.3)	229 (41.3)	567
Islam	459 (56.1)	325 (58.6)	784
Traditional worshiper	21 (2.6)	1 (0.2)	22
Status of residence			
Owned	209 (25.6)	118 (21.3)	327
Not owned	597 (73.0)	414 (74.6)	1011
Others	12 (1.5)	23 (4.1)	35
Gravidity status			
Prime-gravid	275 (33.6)	–	275
Multi-gravid	543 (66.4)	555 (100.0)	1098
Parity			
No child	275 (33.6)	–	275
One child	250 (30.6)	135 (24.3)	385
Two Children	165 (20.2)	174 (31.4)	339
Three or more children	128 (15.6)	246 (44.3)	374
HIV status			
Positive	12 (1.5)	8 (1.4)	20
Negative	603 (73.7)	442 (79.6)	1045
Not known	203 (24.8)	105 (18.9)	308
Blood group			
A	290 (35.5)	184 (33.3)	474
B	133 (16.3)	131 (23.7)	264
AB	51 (6.2)	66 (12.0)	117
O	342 (41.9)	171 (31.0)	513
Genotype			
AA	574 (70.4)	366 (65.9)	940
AS	190 (23.3)	122 (22.0)	312
AC	41 (5.0)	49 (8.8)	90
SS	10 (1.2)	18 (3.2)	28

With regards to the clinical characteristics of respondents, about a third of the pregnant women were primegravida (33.6%) while the rest were multigravidae (66.4%). There were about 1.5% of pregnant women and 1.4% among the non-pregnant mothers of children aged under 5 years who self-reported that they HIV positive. Also, 24.8% and 18.9% of the pregnant women and non-pregnant mothers of children aged under 5 years did not know their HIV sero-status, respectively. With regards to the blood group of the respondents blood group ‘AB’ was less common (6.2% vs 12%, in pregnant and non-pregnant mothers of children aged under 5 years, respectively). Conversely, the predominant genotype was ‘AA’ (pregnant women: 70.4% vs non-pregnant mothers of children aged under 5 years: 65.9%) followed by ‘AS’ (pregnant women: 23.3% vs non-pregnant mothers of children aged under 5 years: 22%), ‘AC’ (pregnant women: 5% vs non-pregnant mothers of children aged under 5 years: 8.8%) and ‘SS’ (pregnant women: 1.2% vs non-pregnant mothers of children aged under 5 years: 3.2%).

### Knowledge about the causes, symptoms and prevention of malaria

Table 2 shows the distribution of variables related to knowledge about malaria disaggregated according to maternal grouping. There was a low proportion of respondents who were not aware of malaria, less than one-tenth among the pregnant women (7%) and even lower among non-pregnant mothers of children aged under 5 years (2.9%). and this was statistically significant, p < 0.05. Almost half proportion of both the pregnant and the non-pregnant mothers of children aged under 5 years did not have knowledge on the breeding sites of mosquitoes (47.1% vs 49.7%, respectively), however this finding was not significant (p > 0.05). Majority of the participants had low knowledge of malaria symptoms and was only able to identify a maximum of 2 or less symptoms of malaria (74% among pregnant mothers and 69% among non-pregnant mothers of children aged under 5 years), the difference in the proportion was on the edge of being statistically significant with p = 0.051. Across both maternal groups, about a third of the respondents reported insecticide treated nets (ITN) as common method of malaria prevention. Similarly, another one-third reported insecticide spray as common prevention methods for malaria. The proportion which reported the correct prevention knowledge for malaria to include ITN, environmental sanitation and chemotherapy such as artemisinin-based combination therapy (ACT), were 39.6% among the pregnant women and 54.2% among non-pregnant mothers of children aged under 5 years, p < 0.001.

**Table 2:** Respondents awareness and knowledge of malaria

	Pregnant women n (%)	Non-pregnant mothers of children aged under five years n (%)	Total N (1373)	Chi square value	p value
Awareness about malaria					
Yes	759 (93.0)	539 (97.1)	1298	11.028	0.001
No	57 (7.0)	16 (2.9)	73		
Causes of malaria					
Mosquito	697 (85.2)	480 (86.5)	1177	12.312	0.031
Contaminated food	8 (1.0)	8 (1.4)	16		
Living in dirty environment	34 (4.2)	32 (5.8)	66		
Too much sunlight or heat	4 (0.5)	5 (0.9)	9		
Don’t know	66 (8.1)	22 (4.0)	88		
Stress	9 (1.1)	8 (1.4)	17		
Correct knowledge on cause of malaria					
Mosquito bites	697 (85.2)	480 (86.5)	1177	0.442	0.506
Causes not mosquito bites	121 (14.8)	75 (13.5)	196		
Breeding sites of mosquitoes					
Stagnant water	433 (52.9)	279 (50.3)	712	0.940	0.332
Other sites/factors not related to breeding sites	385 (47.1)	276 (49.7)	661		
Symptoms of malaria					
Cold	281 (34.5)	254 (45.8)	535	18.122	0.000
Fever	369 (45.1)	265 (47.7)	634	0.926	0.336
Headache	350 (42.8)	330 (59.5)	680	36.767	0.000
Vomiting	75 (9.2)	57 (10.3)	132	0.462	0.497
Weakness	167 (20.4)	69 (12.4)	236	14.805	0.000
Dizziness	36 (4.4)	25 (4.5)	61	0.008	0.927
Nausea	6 (0.7)	6 (1.1)	12	0.461	0.497
Loss of appetite	42 (5.1)	31 (5.6)	73	0.134	0.714
Bitter mouth taste	56 (6.8)	38 (6.8)	94	0.000	0.999
Convulsion	7 (0.9)	9 (1.6)	16	1.684	0.194
Diarrhoea	6 (0.7)	7 (1.3)	13	0.982	0.332
Joint pain	54 (6.6)	47 (8.5)	101	1.691	0.193
Coloured/yellowed eye	10 (1.1)	5 (0.9)	15	0.316	0.574
Coloured/yellowed urine	6 (0.7)	1 (0.2)	7	0.316	0.574
Knowledge on symptoms of malaria					
0–2 correct symptoms	525 (74.0)	358 (69.0)	883	3.812	0.051
Three correct symptoms or more	184 (26.0)	161 (31.0)	345		
When does mosquitoes feed					
Wrong knowledge as other times	300 (36.7)	240 (43.2)	540	5.979	0.014
Correct knowledge as night	518 (63.3)	315 (56.8)	833		
Malaria preventive methods					
Insecticide spray	305 (37.3)	205 (36.9)	510	55.885	0.000
Chemoprophylaxis	15 (1.8)	11 (2.0)	26		
Any bed net	44 (5.4)	8 (1.4)	52		
Insecticide-treated nets	289 (35.3)	274 (49.4)	563		
Drinking traditional concoction	5 (0.6)	1 (0.2)	6		
Keeping environment neat and clean	20 (2.4)	16 (2.9)	36		
Others	140 (17.1)	40 (7.2)	180		
Malaria prevention knowledge					
Has correct knowledge on chemotherapy, insecticide-treated nets and environmental sanitation	324 (39.6)	301 (54.2)	625	28.520	0.000
Does not have correct knowledge	494 (60.4)	254 (45.8)	748		

There was no significant difference in malaria knowledge score between pregnant women and non-pregnant mothers of children aged under 5 years in the study (Table 3). There was also no statistical difference in knowledge score between the age groupings of the respondents. Significantly, knowledge on malaria was higher among respondents who were of the lower middle class (4.10 ± 1.28) and lower upper class (4.10 ± 1.26) than the lower class (3.73 ± 1.66), F = 4.43, p < 0.001. Knowledge score was also highest among the never married women (4.31 ± 1.52, F = 30.2, p < 0.001) compared with the other like the married group (1.08 ± 1.26, F = 30.2, p < 0.001). Educational status of the mothers was also associated with knowledge of malaria as mothers who had secondary (4.07 ± 1.28) and tertiary education (4.20 ± 1.18) as their highest educational qualification showed significantly better knowledge about malaria than those with no formal education (3.38 ± 1.84) and primary education (3.38 ± 1.79), F = 16.80, p < 0.001. The clinical characteristics of the women such as gravidity status, HIV status, blood group and genotype showed significant relationship with malaria knowledge (Table 3). Women with more than a single child had better knowledge of malaria. Respondents whose HIV sero-status, was either positive (4.35 ± 0.88) or negative (4.14 ± 1.21) had higher mean knowledge score about malaria than those who did not know their HIV status (3.63 ± 1.71), p < 0.001.

**Table 3:** Association between selected socio-demographic and clinical characteristics with respondents’ knowledge on malaria

	Mean	Standard deviation	Number	F-statistic	p value
Maternal grouping					
Pregnant women	3.80	0.47	292	2.48^a^	0.116
Mothers of under-five	3.87	0.50	171		
Age group					
< 24	4.12	1.27	207	1.506	0.222
25–34	3.98	1.41	954		
35+	4.13	1.16	212		
Socio-economic status					
Lower class	3.73	1.66	202	4.431	0.004
Lower middle class	4.10	1.28	219		
Lowe upper class	4.10	1.26	833		
Upper class	3.95	1.38	115		
Marital status					
Never married	4.31	1.52	42	30.725	0.000
Married	4.08	1.26	1261		
Separated/widowed	2.83	2.13	70		
Education					
No formal education	3.38	1.84	97	16.808	0.000
Primary	3.38	1.79	81		
Secondary	4.07	1.28	709		
Tertiary	4.20	1.18	486		
Gravidity status					
Prime-gravida	3.45	1.74	275	64.18^a^	0.000
Multigravida	4.17	1.20	1098		
HIV status					
Positive	4.35	0.88	20	17.691	0.000
Negative	4.14	1.21	1045		
Not known	3.63	1.71	308		
Blood group					
A	4.04	1.37	474	7.294	0.000
B	3.70	1.56	264		
AB	4.06	1.26	117		
O	4.17	1.21	513		
Genotype					
AA	4.10	1.26	940	2.9	0.034
AS	3.86	1.60	312		
AC	3.89	1.35	90		
SS	3.86	1.24	28		

^a^t-test

Table 4 presents the post hoc analysis performed to show where the difference in mean for sub-groups significantly associated with knowledge score in Table 3 occurred. The post hoc analysis also shows significant association between selected socio-demographic and clinical characteristics with patients’ knowledge on malaria (Table 4). There was significant association between socio-economic status of the women in the study and their malaria knowledge score. The significant differences were between the lower class and the lower middle class; also between lower class and lower upper class. There was also significant difference between: women who had primary education compared to women who had secondary and tertiary education; women who had secondary education compared to women who had no formal and primary education.

**Table 4:** Post Hoc analysis for significant association between socio-demographic and clinical characteristics with knowledge on malaria score

		Mean difference (I − J)	Sig.	95% confidence interval	
				Lower bound	Upper bound
(I) Socio-economic status	(J) Socio-economic status				
Lower class	Lower middleclass	− .3678*	0.026	− 0.7041	− 0.0314
	Lower upper class	− .3682*	0.003	− 0.6386	− 0.0978
	Upper class	− 0.2152	0.516	− 0.6179	0.1876
Lower middleclass	Lower class	0.3678*	0.026	0.0314	0.7041
	Lower upper class	− 0.0004	1	− 0.2622	0.2614
	Upper class	0.1526	0.756	− 0.2444	0.5497
Lower upper class	Lower class	0.3682*	0.003	0.0978	0.6386
	Lower middleclass	0.0004	1	− 0.2614	0.2622
	Upper class	0.153	0.66	− 0.19	0.4960
Upper class	Lower class	0.2152	0.516	− 0.1876	0.6179
	Lower middleclass	− 0.1526	0.756	− 0.5497	0.2444
	Lower upper class	− 0.153	0.66	− 0.496	0.1900
(I) Marital status	(J) Marital status				
Never married	Married	0.2263	0.521	− 0.2614	0.7139
	Separated/widowed	1.4810*	0	0.8742	2.0877
Married	Never married	− 0.2263	0.521	− 0.7139	0.2614
	Separated/widowed	1.2547*	0	0.873	1.6364
Separated/widowed	Never married	− 1.4810*	0	− 2.0877	− 0.8742
	Married	− 1.2547*	0	− 1.6364	− 0.8730
(I) Education	(J) Education				
No formal education	Primary	− 0.0013	1	− 0.5164	0.5139
	Secondary	− .6905*	0	− 1.061	− 0.3200
	Tertiary	− .8140*	0	− 1.1946	− 0.4334
Primary	No formal education	0.0013	1	− 0.5139	0.5164
	Secondary	− .6892*	0	− 1.0906	− 0.2878
	Tertiary	− .8128*	0	− 1.2235	− 0.4020
Secondary	No formal education	0.6905*	0	0.32	1.0610
	Primary	0.6892*	0	0.2878	1.0906
	Tertiary	− 0.1235	0.392	− 0.3251	0.0780
Tertiary	No formal education	0.8140*	0	0.4334	1.1946
	Primary	0.8128*	0	0.402	1.2235
	Secondary	0.1235	0.392	− 0.078	0.3251
(I) HIV status	(J) HIV status				
Positive	Negative	0.2132	0.76	− 0.4951	0.9214
	Not known	0.7201	0.052	− 0.0038	1.4441
Negative	Positive	− 0.2132	0.76	− 0.9214	0.4951
	Not known	0.5070*	0	0.3036	0.7104
Not known	Positive	− 0.7201	0.052	− 1.4441	0.0038
	Negative	− .5070*	0	− 0.7104	− 0.3036
(I) Blood group	(J) Blood group				
A	B	0.3389*	0.006	0.0731	0.6047
	AB	− 0.024	0.998	− 0.3813	0.3333
	O	− 0.1357	0.389	− 0.3562	0.0848
B	A	− .3389*	0.006	− 0.6047	− 0.0731
	AB	− 0.3629	0.072	− 0.7473	0.0215
	O	− .4746*	0	− 0.7367	− 0.2124
AB	A	0.024	0.998	− 0.3333	0.3813
	B	0.3629	0.072	− 0.0215	0.7473
	O	− 0.1117	0.85	− 0.4663	0.2429
O	A	0.1357	0.389	− 0.0848	0.3562
	B	0.4746*	0	0.2124	0.7367
	AB	0.1117	0.85	− 0.2429	0.4663
(I) Genotype	(J) Genotype				
AA	AS	0.2368*	0.037	0.0098	0.4637
	AC	0.21	0.493	− 0.1732	0.5933
	SS	0.2418	0.787	− 0.4244	0.9080
AS	AA	− .2368*	0.037	− 0.4637	− 0.0098
	AC	− 0.0267	0.998	− 0.4423	0.3889
	SS	0.005	1	− 0.6802	0.6903
AC	AA	− 0.21	0.493	− 0.5933	0.1732
	AS	0.0267	0.998	− 0.3889	0.4423
	SS	0.0317	1	− 0.7199	0.7834
SS	AA	− 0.2418	0.787	− 0.908	0.4244
	AS	− 0.005	1	− 0.6903	0.6802
	AC	− 0.0317	1	− 0.7834	0.7199

In the multivariate linear regression analysis to examine the predictors of malaria knowledge, socio-demographic factors including marital status, education, gravidity status and the clinical factor HIV status remained significant with malaria knowledge (Table 5).

**Table 5:** Multivariate linear model of factors associated with knowledge of malaria

	Unstandardized regression coefficient (95% CI)	95% CI		Standard error	Standardized coefficient	t-statistic
		Lower bound	Upper bound			
Age	− 0.004	− 0.018	0.009	0.007	− 0.02	− 0.60
Wealth status	0.03	− 0.051	0.117	0.04	0.02	0.77
Marital status	− 0.47	− 0.724	− 0.205	0.13	− 0.10	− 3.51***
Education	0.16	0.072	0.252	0.05	0.10	3.52***
Gravidity status	0.67	0.474	0.859	0.10	0.20	6.80***
HIV status	− 0.32	− 0.478	− 0.16	0.08	− 0.10	− 3.93***
Blood group	0.04	− 0.014	0.092	0.03	0.04	1.44
Genotype	− 0.08	− 0.175	0.022	0.05	− 0.04	− 1.52
Maternal grouping	− 0.14	− 0.291	0.017	0.08	− 0.05	− 1.75

R^2^ = 0.050, F for change in R^2^ = 2.328, p = 0.011, * p < .05, ** p < 0.01; *** p < 0.001

## Discussion

Nigeria contributes the highest morbidity and mortality rates to the global burden of malaria, accounting for 25% of the global malaria cases and about 24% of global malaria-related deaths [1]. Thus, the initiative to study maternal knowledge on malaria prevention was essential in understanding the extent and impact of malaria programmatic efforts in malaria control. Women serve as role models for their families in raising awareness and participating in malaria prevention and control [18]. They are also responsible for home-based management of malaria for themselves when pregnant and among children aged under 5 years in the home [19]. In this study, findings revealed obstacles to effective malaria control despite high awareness of malaria as an illness which has been previously reported in studies conducted in South Western Nigeria [20], Northern Central Nigerian [21] and as confirmed in this study (93% among pregnant women and 97% among mothers of young children). There were knowledge gaps on; breeding sites for the vectors that transmit malaria, symptoms of malaria and malaria prevention measures. According to Killeen [22], level of knowledge on mosquito behavioural pattern (biting and resting times) and breeding sites has been associated with the severity of malaria. Killeen further explains that elimination of malaria from most endemic regions of the tropics requires vector control strategies that address residual transmission by deliberately targeting the mosquito behaviours which enable it [22].

In relation to the knowledge on malaria symptoms and preventive measures by respondents in this study, about 60% of pregnant women and 46% of non-pregnant mothers of young children did not have correct knowledge on malaria prevention. Further, there were 26% of pregnant mothers and 31% of the non-pregnant mothers of young children who correctly reported more than 3 clinical symptoms of malaria. Similar studies conducted in rural South West Nigeria [14], North Central Nigeria [9] and Burkina Faso [18] also showed low knowledge on malaria prevention measures. Conversely, the study by Singh et al. showed that high knowledge about malaria symptoms and prevention measures (90%) however; this knowledge was poorly reflected in practice (16%) [13]. Misconceptions about causes of malaria in this study although reported by few respondents include living in dirty environment, eating contaminated food, stress, and exposure to sunlight. Some studies in Nigeria and parts of Africa have also reported spurious causes of malaria such as staying for long in the sun and drinking bad water among other misconceptions on malaria [11, 21, 23, 24]. Overlapping knowledge on malaria causes, key symptoms, and prevention was observed between pregnant women and the non-pregnant mothers of children aged under 5 years in this study. In some aspects of malaria prevention, higher proportion of pregnant women was less knowledgeable about malaria, compared with the mothers of young children and vice versa. However, the differences in malaria knowledge on preventive measures between the maternal groups were not significant from the analysis of variance performed.

Level of knowledge on malaria was associated with; socio-demographic factors such as marital status, education and clinical factors like gravidity and HIV status of the mothers. Good malaria knowledge was associated with higher level of educational status of the women. In previous studies, educational status has been linked with good health awareness and health-seeking behaviour for the child [23, 25], and also improved knowledge on malaria and prevention among mothers [9, 18, 26]. Such association according to Fana et al. stresses the role education could have on the overall success in malaria control programmers in a region [26]. Another important finding was that respondents who knew their HIV status had a good knowledge of malaria compared with those who did not know their HIV status. Further, those who were HIV positive had better malaria knowledge when compared with both those were HIV negative and those who did not know their HIV status. The high knowledge of malaria among HIV positive respondents in the study might be due to the awareness of the high risk of acquiring opportunistic infections. For instance, knowledge of HIV status as reported by the study respondents reflects a higher awareness of their health status. This agrees with finding from study in Uganda by Katrak et al. where a > sixfold lower risk of infection with malaria parasites among HIV-infected participants with an undetectable viral load was seen when compared to HIV-uninfected participants [27]. Possible explanation could be because individuals who knew their HIV status tend to have good health-seeking behaviour and knowledge on malaria compared with those who do not know their HIV status.

Although the study investigated the knowledge of malaria prevention and control, and sought to find the socio-demographic and some clinical factors associated with malaria knowledge this study did not investigate the programmatic factors that may influence the knowledge of the respondents on malaria and would like to recommend this for future studies. Limitations of this study include recall bias on account of information provided by the respondents. Since the study population was hospital-based, another bias related to the limitation of this study is selection bias because this hospital based study population could have been more knowledgeable than similar population if recruited from the community. Though these limitations, this study has implications for control programmes given the findings, which highlights the knowledge gaps requiring urgent interventions targeted at mothers.

## Conclusion

This study has demonstrated that pregnant women and mothers of children under 5 years are aware of malaria, but still lack comprehensive knowledge about the disease. Many mothers know some important symptoms of malaria such as fever, cold and headache. There was also some level of misconception about malaria, which needs to be totally debunked by intensifying education about malaria among mothers who are either pregnant and or caring for young ones who are more vulnerable to malaria disease. Education as a socio-demographic factor was an important predictor knowledge of malaria among mothers and so government policies should be geared towards improving citizens ‘educational statuses in order to reduce the burden of the disease in the country, especially among the most vulnerable population. Mothers need to be educated about the importance of a better health-seeking behaviour and awareness about their health status. Nigeria’s malaria strategic plan should to ensure that the knowledge cleft on malaria prevention and treatment needs to be addressed. This insight will help the policy makers to implement continuous strategic intervention including health awareness and educational programs to attain 2030 malaria goals.

## Abbreviations

ACT: artemisinin-based combination therapy
BREC: biomedical research ethics committee
GTS: global technical strategy
HIV: human immunodeficiency virus
IDI: in-depth interviews
IPT: intermittent preventive treatment
IPTp: intermittent preventive treatment of malaria in pregnancy
ITN: insecticide treated nets
IVM: integrated vector management
KAP: knowledge, attitudes, and practices
LLIN: long-lasting insecticide-treated nets
NMSP: national malaria strategic plans
SPSS: statistical package for social sciences
WHO: World Health Organization
